# Arabicization or Englishization of higher education in the Arab world? Controversies, policies and realities

**DOI:** 10.3389/fpsyg.2023.1093488

**Published:** 2023-02-15

**Authors:** Muhammad M. M. Abdel Latif, Majed M. Alhamad

**Affiliations:** ^1^Faculty of Graduate Studies of Education, Cairo University, Giza, Egypt; ^2^Department of Studies of Arabic (L2), College of Language Sciences, King Saud University, Riyadh, Saudi Arabia

**Keywords:** EMI, Arabicization, Englishization, language policy, higher education internationalization, second language learning

## Abstract

Though there have been many calls for the Arabicization versus Englishization of higher education in the Arab world, no previous work seems to have comprehensively covered these calls and their outcomes in the region. In this paper, the authors review literature on the following four main pertinent issues: (a) the arguments for and against the Arabicization versus Englishization of higher education in the Arab world; (b) Arabicization attempts of higher education; (c) current Englishization policies and orientations of Arab higher education systems; and (d) realities of EMI practices in Arab universities. It is concluded that higher education Arabicization movements in the Arab world have not brought about their desired outcomes due to some barriers, whereas Englishization policies and practices have increasingly expanded in the region in the last three decades. The paper ends with discussing the implications of the review.

## 1. Introduction

In the last few decades, the Englishization of higher education has become a growing international phenomenon. In non-Anglophone countries worldwide, higher education institutions have paid increasing attention to offering students academic programs in which English is used as a medium of instruction. This international orientation in adopting English medium instruction (EMI) policies has stemmed from a number of forces. Overall, these include: recognizing the importance of English as an international academic and workplace language; promoting the international reputation of higher education systems and in turn attracting local and overseas students; meeting the needs of students receiving bilingual education at the pre-university stages; and coping with the nature of teaching materials (Coleman, 2006; Doiz et al., 2013; Knight, 2013; Macaro et al., 2018; Galloway, 2020).

While many countries and international societies are increasingly oriented towards higher education Englishization policies, the case is rather different in Arab countries. The Arab world consists of 22 countries, 19 located in Western Asia and Northern Africa, two in the Horn of Africa (Djibouti and Somalia), and one in the Indian Ocean (the Comoros). The 22 countries share some cultural and linguistic characteristics. These Muslim majority countries use Arabic as their official language. English is the main foreign language in all Arab countries with the exception of Algeria, Djibouti, Morocco, the Comoros, and Tunisia which depend on French as a second language.

There have been many controversies over higher education Englishization policies in Arab countries as they were countered by calls for the Arabicization of curricula and teaching. Such controversies partially account for the fact that the adoption of Englishization polices in higher education institutions varies from one Arab country to another. Though Arabicization and Englishization issues have long been debated in the Arab world, no previous work seems to have adequately addressed them and their related outcomes in Arab higher education institutions. In an attempt to address this gap, this paper reviews the literature on the controversies over the Arabicization or Englishization of higher education institutions in Arab countries, and the policies and realities of higher education Arabicization versus Englishization. Specifically, the paper deals with the following issues:

- Arguments for and against the Arabicization versus Englishization of higher education;
- Arabicization attempts of higher education in the Arab world;
- Current Englishization policies and orientations of Arab higher education systems;
- Realities of EMI practices in Arab universities.

The authors try to cover these issues drawing on synthesizing relevant literature and published and non-published research findings. The aim is to provide readers with a comprehensive picture about the debates, policies and realities of Arabicization versus Englishization in Arab world universities. Thus, this review could be of potential significance to those interested in language policies in Arab countries and in EMI practices in the region.

## 2. Arguments for and against the Arabicization vs. Englishization of higher education

There have been a lot of controversies over the Arabicization versus Englishization of higher education in the Arab world. The opponents and proponents of each orientation have their own justifications. The main reason for the Arabicization movement is strengthening the Arab and Islamic identity and culture. Englishization of higher education may be regarded by some as a policy for marginalizing Arabic as an academic language and devaluing the Arab cultural and Islamic identity (Hanafi and Arvanitis, 2014; Solloway, 2016; Selvi and Yazan, 2017). In other words, it is viewed as a form of linguistic imperialism (Phillipson, 1992). It can be generally noted that the active Arabicization movements in some Arab countries, for instance in Sudan and Syria, was stimulated by a major political change. As Zughoul (2000) concludes, Arabicization is a more politically-than technically-driven process.

Arabicization of higher education is also viewed as a way for preserving the Arabic language. For example, Al-Issa (2017) views that EMI policies in the United Arab Emirates could lead to the endangerment of Arabic literacy which is gradually losing ground. This conclusion is supported by Masri (2019, 2020) research findings that as a result of EMI policies, Arab students in Emirati universities have lost their faith in Arabic as an academic language and have had a decline in their Arabic proficiency levels.

Another main factor associated with the calls for using Arabic as a medium of instruction in Arab universities is to facilitate students’ learning and understanding of content materials. Proponents of Arabicization hold the opinion that many Arab students do not have the English proficiency levels needed for meeting their study requirements, and thus using Arabic as a medium of instruction could save time and efforts (Hanafi and Arvanitis, 2014). In Egypt, for instance, Sabbour et al. (2010) found that the larger number of medicine students have difficulties in studying their courses in English. Likewise, Alhamami and Almelhi (2021) study revealed that healthcare students in Saudi Arabia prefer to study their majors in Arabic as it enhances their understanding and improves their academic achievement. In the Omani context, Al-Masheikhi et al. (2014) found that though university students are aware of the status of English as the global language of science and technology, more than half of them opt to use Arabic as a medium for studying their majors. On the other hand, research also indicates that the faculty members teaching science majors to Saudi students like to use Arabic as their medium of instruction despite the EMI policy adopted by Saudi universities (Alhamami, 2015).

A third key factor stimulating the calls for Arabicization relates to considering the nature of some core subjects, and the majors students study. For example, Sabbour et al. (2010) reported that medicine students in Egypt prefer to do some clinical study dimensions in Arabic rather than English. Their faculty members also view that depending on EMI in some medicine courses creates a gap between the teaching language (i.e., English) and the language used in actual practices in medical settings (i.e., Arabic). As for the potential incompatibility of EMI policies with particular university majors, it may apply more to some social sciences and humanities majors. This case is clear, for instance, in Qatar University which replaced the EMI policy in 2012 with the Arabicization of curricula and teaching in its faculties of law, international affairs, mass communication, and business and economics. It is worth to note, however, that Ellili-Cherif and Alkhateeb (2015) study indicates a contradiction in Qatar university students’ attitudes towards such Arabicization policies. While they prefer using Arabic as a medium of their instruction, they are aware that this could jeopardize their employability.

The forces driving the Englishization of higher education in the Arab world are similar to those given in the introductory sections. These forces include: fostering the marketization of higher education institutions (Hanafi, 2011), realizing the role of English as a global communication language in educational and workplace settings (Al-Issa, 2017; Denman and Al-Mahrooqi, 2019), enhancing the employability of university graduates (Hanafi and Arvanitis, 2014), facilitating immediate transmission and knowledge of new scientific and technological developments, i.e., *via* the materials written in English (Al-Saadat and Sheikh Al-Shabab, 2005), improving university ranking (Belhiah and Abdelatif, 2016), and promoting national modernization (Al Zumor and Abdesslem, 2022).

Arguably, the factors accounting for the Englishization of higher education in the region have also stemmed from the obstacles to Arabicization of applied sciences in particular. Zughoul (2000) identifies the following three main obstacles to Arabicization movements: (a) the counterproductivity of using Arabic in teaching highly technical applied sciences in which coining and using terminological terms can be chaotic; (b) the lack of source materials, books and journals written in Arabic; and (c) the need for more regular and harder work on translating specialized terminology in scientific majors.

Due to the factors and driving forces mentioned above, the Arab world has recently seen more orientations towards the Englishization rather than Arabicization of higher education. In Saudi Arabia, for instance, English seems to be in the process of being nativized and having its own emergent variety (Mahboob and Elyas, 2014). In the United Arab Emirates, there has been an overwhelming trend in adopting EMI policies in most higher education institutions (Al-Issa, 2017). For example, this trend can be noted in the contents of Emirati universities websites, and the requirements of vacant faculty positions. Some other relevant major developments have occurred in other Arab countries. This is what will be discussed in a later section.

## 3. Arabicization attempts of higher education in the Arab world

Some Arab countries have adopted Arabicization policies in their higher education institutions. The clearest Arabicization attempts in the region have perhaps been in Syria, Sudan and to a less extent in Jordan. Below is a brief description of these attempts and their outcomes.

Syria has made pioneering efforts in the Arabicization of applied sciences in its universities. While these efforts date back to the 1910s–1920s, the Arabicization movement was accelerated in the late 1950s after Syria got its independence. The strength of the Arabicization movement in the Syrian universities during the 1990s is clearly described by Badinjki (1994):

Arabic is the only language of instruction, and all textbooks and references are in Arabic. Each university has its own printing office that supplies it with its needs. In the printing office of the University of Aleppo alone, for example, 1,680 textbooks and academic references have so far been produced all in Arabic. … The method adopted by the university staff in introducing new terms followed certain steps. In order of priority, they arc: a) searching in old Arabic books for a term with an equivalent meaning; b) searching for an old word or term with an analogous meaning that, with a little modification, can be adopted, c) searching for a new word meaning within the accepted rules of derivation; and d) transliteration when translation and derivation arc impossible. (p. 112)

Despite the above-mentioned status, Badinjki refers to a number of obstacles hindering the Arabicization of sciences in Syrian universities, including the irregularities of institutional translation efforts of science references and the lack of the incentives for them, the rapid developments in sciences and technology, and differences in translating scientific terms within the Arab world.

It is worth to note that very scarce recent works seem to have been published on current medium instruction language practices in Syrian universities. There is also a dearth of the recent empirical research on the evaluation of the Arabicization phenomenon in this setting. Evaluating the Syrian experience in the Arabicization of higher education, Balla (1991) concludes that it is far from perfect as its demerits outweigh merits. In a more recent observation-based study about the use of Arabicization and Arabic expanding techniques in Jordan University of Science and Technology (in Jordan) and the University of Damascus (in Syria), Al-Asal and Smadi (2012) found that Arabicized scientific terms were less frequently used in the Syrian university than in the Jordanian one. This suggests that currently there seems to be more use of English than Arabic when referring to scientific terms in Syrian universities.

In Sudan, the governmental efforts towards the Arabicization of higher education occurred in the early 1990s after Al-Bashir came to power. Al-Bashir’s government issued the 1990 Higher Education Act which aimed to Arabicize and Islamize the Sudanese higher education system. According to Gasim (2010), the curriculum Arabicization movement in Sudan, known as ‘the higher education revolution’ (p. 53), represented a political orientation and was incepted in a hasty decision taken was without considering stakeholders’ views and training needs or the availability of Arabic curriculum materials; this is why it had many pitfalls. Other related studies concerned with the Sudanese experience indicate that not many gains have been obtained from this Arabicization movement (e.g., Suliman, 2004). Alhassan (2022) goes further by viewing that such Arabicization policies of higher education in Sudan have failed and have been detrimental to teachers and students.

In addition to these country-specific governmental initiatives, there have been efforts made by individual bodies towards Arabicizing scientific terms in the Arabic world. Specifically, these efforts have been made by Arabic language academies in some Arab countries. The Jordan Academy of Arabic Language has perhaps the most notable efforts in this regard. It has initiated a huge project that aimed at the Arabicization of higher education sector through localizing scientific terminology and translating scientific textbooks, and Arabicizing scientific notations (Al-Ajrami, 2015). Zughoul (2000) gives a brief idea about the contributions of this initiative to the Arabicization of instruction at Jordanian universities:

A project worth pointing out as an example in connection with the language of higher education is the ambitious and large scale project of translating the most widely used science textbooks in the areas of Mathematics, Biology, Physics, Algebra, Geology and Chemistry undertaken by The Jordanian Academy of Arabic. The textbooks for the first university year were completed and field tested while work is still going on the textbooks for the rest. (p. 8)

In spite of these efforts, empirical studies on the acceptance of Arabicized (i.e., translated) scientific terms in Jordan have reported mixed results (Al-Haq and Al-Essa, 2016; Yaseen, 2016). Thus, this Arabicization attempt has not gained much acceptance in Jordan; Zughoul (2000) concludes that it has not been successful.

The above three examples clearly show that Arabicization policies and movements in some Arab countries have not brought about their desired outcomes. These have generally been individual and country-specific efforts. Badinjki (1994) states that “the efforts expended by one single country, no matter how great, cannot cover all fields of knowledge” (p. 12). More than two decades ago, Bou Jaoude and Sayah (2000) argue that the discussions about the medium of instruction in Arab higher education institutions is like an ideological debate in which there is a realistic call for the need for communicating scientific information through English and a dreamy one for drawing upon Arabicized science education. In fact, Bou Jaoude and Sayah's (2000) view applies particularly to science majors whose Arabicization seems to be beyond reach.

## 4. Current Englishization policies and orientations of Arab higher education systems

As indicated above, growing attention has recently been given to the Englishization policies of higher education in the Arab world. It is noteworthy, however, that EMI policies of higher education differ from one Arab country to another. The historical beginnings of such polices also vary. For example, English has been used as the language of teaching at Kasr Al Ainy Medical School (currently Faculty of Medicine, Cairo University) in Egypt towards the end of the 19th century (Saleem, 2021), but only in recent years it has started to gain ground as a medium of instruction in Tunisian and Moroccan universities (see Badwan, 2022; R’boul, 2022, respectively). At present, there are different types of EMI universities and university programs in the region.

International branch campuses (IBCs) – operated by international universities for expanding global outreach and student exchange – are now found in some Arab countries. The United Arab Emirates hosts the largest number of IBCs in the Arab world (Rensimer, 2021). It has campuses for international universities such as the University of Birmingham, Heriot-Watt University, the University of South Wales, and the University of Bradford. Likewise, Qatar has now campuses for international higher education institutions such as Carnegie Mellon University, Texas A & M University and Texas A & M University (see Hanada, 2013). Recently, Egypt has started to attract IBCc to its New Administrative Capital. Coventry University and the University of Hertfordshire are among the IBCs founded in Egypt (see Lane, 2018). These IBCs adopt EMI policies, recruit multi-national faculty members, and teach all their curricula in English, the shared language of faculty members and students.

Foreign universities represent another type of EMI higher education institutions in the Arab world. They are not IBCc but Arab world-based universities established by foreign countries or organizations. The well-known examples of these universities include: the American University in Cairo, the American University in Beirut, American University in Sharjah, the British University in Dubai, the British University in Egypt, and the German University in Cairo. The EMI policies and practices in such foreign universities resemble those in IBCs. Both university types admit students based on a minimum score on an international standardized test (e.g., IELTS, TOEFL), and provide students with regular English language support. That is why the students who join foreign universities and IBCc are likely to have better English proficiency levels than their peers in the other types of EMI universities and/or university programs in the Arab world.

The other higher education institutions in the Arab world are national or local ones established by state governments or mainly owned by individuals from the same country. These national universities are either public or private, but the larger number of them in most Arab countries is public. Some national Arab universities, mainly private ones, adopt EMI policies in all their programs. This can be noted, for example, at Nile University, Zewail City of Science and Technology (Egypt), Khalifa University, United Arab Emirates University (United Arab Emirates), Gulf University for Science & Technology (Kuwait), and Prince Sultan University (Saudi Arabia). Most of these national EMI universities have lower language-related admission requirements and less intensive language courses than those in IBCs or foreign universities.

Other national universities in the region are basically Arabic medium instruction higher education institutions, but they have implemented EMI policies only in applied science majors such as medicine, engineering, chemistry, physics, biology and mathematics, and– in some cases-in particular social sciences and humanities fields such as business administration, mass communication and politics. The language-related admission requirements and language support of EMI students in these Arabic medium universities vary considerably. Admission requirements could be a minimum score on a high school English exam or on a local English test. As for the language support, it is generally limited and could take the form of an English-for-specific-purposes (ESP) course taught for one or two terms [see relevant findings in Abdel Latif (2023) and Belhiah and Abdelatif (2016)].

The last three decades have seen major developments in establishing the above-mentioned EMI universities and programs in all regions of the Arab world (see Hanada, 2013; Lane, 2018; Abdel Latif, 2023). This wider adoption of EMI policies is not limited to the Arab Gulf states and some Arab countries such as Egypt, but it also encompasses Maghreb countries (i.e., Algeria, Morocco and Tunisia) in which French is used as a second language. For example, there have been some endevours to shift from French to English as the language of teaching in Algerian higher education institutions. Stakeholders’ attitudes to such transition have been found positive (Kaddari and Mazouzi, 2022; Kadi, 2022; Maarouf and Lamouri, 2022). While French is the medium of instruction in most academic programs in the Tunisian higher education system, some universities in the capital city of Tunisia have started to use EMI instead (Badwan, 2022). On the other hand, English has recently gained greater ground in Moroccan universities, and there is now wide support for replacing French with English in them (Jebbour, 2021). According to R’boul (2022), postgraduate students in Morocco are encouraged to write their research reports in English and publish in ranked journals written in English, and a number of Moroccan private universities have embraced EMI policies such as Al-Akhawayn University and the International University of Rabat. Additionally, Morocco hosts some IBCs for British universities such as the University of Sunderland and Cardiff Metropolitan University. Research evidence also indicates that both Moroccan university students and teachers realize the importance and necessity of using EMI (Belhiah and Abdelatif, 2016; R’boul, 2022).

As indicated above, the Englishization of higher education systems and programs in many Arab countries is on the rise. This movement is motivated by forces almost identical to those found in other world regions. Given the realities of such Englishization movement and its driving forces along with obstacles mentioned earlier to the Arabicization of higher education (particularly in relation to applied sciences majors), it is important now to focus on how to optimize EMI practices in the Arab world.

## 5. Realities of EMI practices in Arab universities

Due to the different types of EMI universities and programs in the Arab world, it is expected to see variance in EMI practices across the region. EMI practices in IBCs and foreign universities in the Arab world seem to have a fewer challenges as compared to those in national universities. An indication of this is that the vast majority of published research on EMI challenges in the region is concerned with this latter university type rather than the former one. Abdel Latif (2023) concludes that EMI practices in IBCs and foreign universities in Egypt do not appear to have teacher-related challenges due to the fact that most faculty members recruited in them are either native speakers of English or Egyptians with PhD degrees from a Western university. Therefore, they are able to deliver EMI classes easily. However, writing seems to be a problematic language area to students attending this university type (Pessoa et al., 2014; Arrigoni and Clark, 2015).

On the other hand, research has shown some realities of EMI practices in Arab national/local universities. A main dimension in these practices is content teachers’ dependence on translanguaging, i.e., switching between English and Arabic. This has been revealed by many research reports about EMI practices in, for instance, Egypt (Abdel Latif, 2023), Libya (Tamtam et al., 2013), Oman (Al-Masheikhi et al., 2014), Somalia (Ohirsi et al., 2022), Sudan (Alhassan et al., 2021), United Arab Emirates (Belhiah and Abdelatif, 2016), and Yemen (Laban, 2008; Zanke and Hasan, 2014). In fact, translanguaging is not a recent phenomenon in the EMI programs in Arab universities. More than four decades ago, an early note was made by Bending (1976) about EMI content teachers’ translanguaging at Egyptian universities; Bending states:

English remains the medium of instruction within university faculties of medicine, dentistry, veterinary studies, engineering and certain other scientific subjects…[P]rofessors at the universities tend to use a mixed medium combining English technical terminology linked together by colloquial [Egyptian] Arabic. (pp. 316–317)

EMI content teachers’ dependence on translanguaging in Arab universities could be mainly attributed to their consideration of students’ low language abilities. In the Qatari context, Hillman et al. (2019) found that content teachers resort to translanguaging even in an IBC university to build rapport, create an inclusive classroom atmosphere, scaffold students’ learning, and give classroom instructions.

Research has revealed that students attending EMI programs in national Arab universities have a number of language problems. While some studies have referred generally to Arab EMI students’ low English proficiency levels (e.g., Al-Masheikhi et al., 2014; Alhamami, 2015; Solloway, 2016; Rahman, 2020), others have indicated that a main difficulty students encounter relates to comprehending lecturers delivered in English and understanding English-written study materials. Previous studies have found these skill problems in Egypt (Sabbour et al., 2010), Saudi Arabia (Shamim et al., 2016), Sudan (Alhassan et al., 2021), Tunisia (Badwan, 2019, 2022), United Arab Emirates (Belhiah and Abdelatif, 2016) and Yemen (Laban, 2008; Al-Kadi, 2018). In an IBC university in Qatar, Pessoa et al. (2014) found that EMI students’ reading difficulties stem from their limited vocabulary, background knowledge, wrong genre expectations, reading stamina, and unfamiliar discourse features. To overcome such problems, students, like their teachers, depend on translanguaging (e.g., Sabbour et al., 2010; Shamim et al., 2016; Solloway, 2016; Alhassan et al., 2021). It seems, therefore, there is a kind of mutual interaction between faculty members’ switching to Arabic and their students’ dependence on translanguaging. In other words, faculty members resort to explaining English written content in Arabic to communicate it more effectively to students and facilitate their understanding, and students alike draw on translation because they see their teachers do the same in classes/lectures or perhaps there is a lack of emphasis on effective English language performance in testing situations (Abdel Latif, 2023).

Some studies have indicated that Arab EMI students have more problems in English writing than in other language areas. This has been confirmed in the studies reported by Ghenghesh et al. (2011), Pessoa et al. (2014), Al-Kadi (2018), Al-Bakri and Troudi (2020), Alhassan et al. (2021), and Al-Bataineh (2021). Al-Bakri and Troudi (2020) study specifically provided a detailed profile of Omani EMI students’ writing difficulties. Their study revealed that Omani EMI students have more difficulties in language-related writing aspects (grammar, spelling and vocabulary, respectively) rather than ideational or organizational features. Regarding the speaking area, some studies have reported that Arab EMI students encounter difficulties in it (e.g., Belhiah and Abdelatif, 2016; Alhassan et al., 2021; Badwan, 2022), but research findings generally indicate that students have more problems in writing, reading comprehension and listening comprehension, respectively.

The language problems Arab university EMI students reported encountering emphasize the need for reconsidering their language-related admission requirements. The issue of language-related admission requirements in Arab university EMI programs has hardly been addressed. Arrigoni and Clark (2015) study is a rare attempt in this regard. In this study, they examined the appropriateness of the IELTS cut-off scores used for placing the American University in Cairo students in remedial courses. Though their study revealed that the cut-off scores were consistent with students’ academic outcomes, and instructor and student evaluations, the scores used for remedial course placement did not match the writing section of the test and the content of the courses.

An issue related to the above one is the lack of appropriate language support for EMI university students in the Arab world. Some university students in the United Arab Emirates do not feel that their high school education was adequate to prepare them for EMI programs (Solloway, 2016). In Qatar universities, any developments in EMI students’ English language levels seem to occur incidentally rather than as a result of direct language instruction (Eslami et al., 2020). In Egypt, in-sessional language support provided to EMI students in national universities is superficial (Abdel Latif, 2023). There is also a need for providing EMI students with adequate in-sessional language support in Omani and Sudanese universities (Al-Masheikhi et al., 2014, Alhassan et al., 2021, respectively). Given these findings, Arab university EMI students are in need of adequate and long-term in-sessional English language support.

Another under-explored issue is Arab university content teachers’ readiness for EMI. Observing some YouTube-recorded lectures of social sciences and humanities EMI study programs in Egyptian public universities, Abdel Latif (2023) concludes that faculty members’ English communicative competence levels vary from one major to another. Though it is generally recognized that some EMI content teachers in Arab universities prefer to depend more on Arabic in their classes as a result of their dysfluent English, the studies highlighting this issue are very scarce. In Iraq, faculty members reported their limited English proficiency levels do not enable them to effectively communicate course content in English (Rahman, 2020). This problem is also noted in the Maghreb countries in which university faculty members depend mainly on French and Arabic as languages of teaching. Badwan (2022) study showed that many Tunisian university faculty members feel unable to teach their major courses using English. More than two thirds of the Algerian faculty members who took part in Kadi (2022) study rate their English proficiency levels as either low or intermediate. Consequently, there is a need for assessing Arab university EMI content teachers’ English proficiency and communication levels, and for providing them with adequate language training and support when necessary.

## 6. Discussion

As indicated above, there have been calls for the Arabicization versus other ones for the Englishization of higher education in the Arab world. The proponents of each medium instruction language orientation have their own reasons. Such calls and attempts have echoed differently. In reality, the calls for Arabicization have not been a success, particularly with regard to scientific majors such as medicine, engineering, chemistry and physics. The Arabicization movements in Syria, Sudan and Jordan have not brought about their desired outcomes due to some factors, including politically-driven education policies, irregularities and individuality of institutional translation attempts, and rapid developments in sciences and technology. In contrast, the last three decades have witnessed increasing orientations in adopting Englishization policies of higher education in many Arab countries, particularly in Arab Gulf states, Egypt, Morocco and Tunisia.

There is a need for optimizing Arabicization and Englishization policies of higher education in the Arab world. Four main factors should be taken into account in this process: (a) preserving the Arabic language and protecting the Arab culture and identity; (b) meeting the requirements of the larger number of workplace environments which require using Arabic and English; (c) considering the status of English as an international academic language; and (d) promoting the internationalization of Arab higher education institutions. In applied sciences majors, Englishization is inventible due to the continuously growing literature written in English and the barriers to translating it.

With regard to social sciences and humanities majors, there is a potential for their Arabicization but students’ expectations and future workplace needs should be taken into account. Therefore, these majors could include some courses taught in English and others taught in Arabic. To meet their Arab and non-Arab students’ various study needs in social sciences and humanities majors, Arab universities could also offer them pure EMI programs and other ones which require the use of Arabic and English as languages of teaching. With these language policies, we could strike a balance between Arabicization and Englishization orientations.

Meanwhile, EMI practices encounter a number of problems in Arab universities. Therefore, there is a need for modifying the language-related admission requirements and fostering pre-sessional and in-sessional English language support practices in these universities. Language-related admission requirements could be modified in a way motivating candidate students to improve their English proficiency levels prior to joining their core academic programs. Alternatively, students could be provided with more intensive English instruction in their foundation programs. Likewise, they also in need for intensive in-sessional language support courses, particularly in writing. Both pre-sessional and in-sessional language courses should focus on familiarizing students with the linguistic features of the genres in their majors.

There is also a need for providing EMI content teachers in Arab universities with language training. As noted above, some reports call for addressing this need in Egypt, Tunisia, and Algeria (Badwan, 2022; Kadi, 2022; Abdel Latif, 2023, respectively). Initial observations also indicate that EMI content teachers in some Arab Gulf countries have their own language support needs. Accordingly, those EMI content teachers with low English proficiency levels should be provided with appropriate language training.

## 7. Conclusion

In the above review, the authors have discussed the controversies, policies and realities of the Arabicization and Englishization of higher education in the Arab world. As implied, Arabicization movements in Arab universities have been encountering some barriers. Due to such barriers, Arabicization policies are unlikely to succeed in most courses taught in applied sciences majors. While these policies seem to be more applicable to social sciences and humanities majors, students’ expectations and workplace requirements necessitate offering them a variety of EMI and Arabic medium instruction programs and courses that meet their varied needs. Meanwhile, there is a need for reforming EMI practices in Arab higher education institutions. This could be accomplished by modifying language-related admission policies, fostering English language support, and improving content teachers’ language performance.

This paper is limited by its nature and scope, i.e., reviewing Arabicization and Englishization issues of higher education institutions in the Arab world. Future works could focus on the similar issues related to the pre-university education in the region. Meanwhile, many EMI issues are still under-explored in Arab higher education institutions. Though the last decade has seen an increase in published relevant studies, EMI has not yet received adequate research attention in many Arab contexts. We hardly know any information about EMI practices in countries such as Bahrain, Djibouti, Iraq, Jordan, Kuwait, Mauritania, Palestine, Somalia, and Syria. There is a scarcity of research on Arab university EMI content teachers’ language performance or needs. Cross-country surveys of language-related admission requirements and/or in-sessional language instruction in Arab universities are still unavailable. No research appears to have examined language interaction and assessment in EMI classes and courses in Arab universities. All these issues need to be tackled in future research.

## Author contributions

ML and MA contributed directly to the conceptualization of the content, literature selection and review, and manuscript writing-up and revision. All authors contributed to the article and approved the submitted version.

## Conflict of interest

The authors declare that the research was conducted in the absence of any commercial or financial relationships that could be construed as a potential conflict of interest.

## Publisher’s note

All claims expressed in this article are solely those of the authors and do not necessarily represent those of their affiliated organizations, or those of the publisher, the editors and the reviewers. Any product that may be evaluated in this article, or claim that may be made by its manufacturer, is not guaranteed or endorsed by the publisher.
